# Personalized Prediction of Cutaneous Melanoma Risk Based on Polymorphism of the Genes Related to Skin Aging, Phenotypic Characteristics, and Sun Exposure in Lithuania

**DOI:** 10.3390/cancers18203323

**Published:** 2026-10-15

**Authors:** Grinvydas Butrimas, Lukas Šemeklis, Renata Paukštaitienė, Aistė Ogulevičiūtė, Gailė Mišeikytė, Dalia Lukšienė, Skaidra Valiukevičienė

**Affiliations:** 1 Department of Skin and Venereal Diseases, Medical Academy, Lithuanian University of Health Sciences (LSMU), LT-50161 Kaunas, Lithuaniaskaidra.valiukeviciene@lsmu.lt (S.V.); 2 Department of Ophthalmology, Medical Academy, Lithuanian University of Health Sciences (LSMU), LT-50161 Kaunas, Lithuania; 3 Department of Physics, Mathematics and Biophysics, Medical Academy, Lithuanian University of Health Sciences (LSMU), LT-44307 Kaunas, Lithuania; 4 Faculty of Medicine, Medical Academy, Lithuanian University of Health Sciences (LSMU), LT-44307 Kaunas, Lithuania; 5 Laboratory of Population Studies, Institute of Cardiology, Lithuanian University of Health Sciences (LSMU), LT-50103 Kaunas, Lithuania

**Keywords:** cutaneous melanoma, risk factors, skin-aging polymorphisms, phenotypic characteristics of skin and iris

## Abstract

**Background/Objectives:** This is the first study in Eastern Europe to integrate skin and iris phenotypic characteristics with selected polymorphisms in genes related to skin aging, melanogenesis and skin pigmentation, and inflammatory regulation in relation to cutaneous melanoma (CM) risk. **Methods:** This case–control study included 180 patients with primary CM identified from the Hospital of the LSMU Kauno klinikos database and 181 controls without a personal history of skin cancer selected from a population-based study in Kaunas. All participants underwent dermatological and ophthalmological examinations and completed questionnaires assessing sun-exposure habits, including severe sunburn history, and Fitzpatrick skin phototype. The total-body number of common melanocytic nevi (CMN) >2 mm in diameter and the number of iris freckles were evaluated using standardized protocols. Five selected genetic polymorphisms, including TYR rs1126809, SLC45A2 rs26722, SLC45A2 rs16891982, IL6 rs1800795, and TNF-α rs1800629, were genotyped. Associations with CM were assessed using multivariable logistic regression. **Results:** We identified three phenotypical factors independently associated with increased odds of CM (OR; 95% CI): a total-body CMN count >15 (7.89; 4.29–14.53), ≥4 iris freckles (3.70; 2.04–6.74), and a history of severe sunburn (2.76; 1.50–5.07). Compared with Fitzpatrick skin phototype IV, phototypes II (0.35; 0.15–0.82) and III (0.28; 0.11–0.70) were associated with decreased CM odds. In the multivariable model the heterozygous SLC45A2 rs16891982 genotype was associated with increased CM odds (6.64; 1.48–29.83), while the IL6 rs1800795 Mono G genotype revealed an inverse association with CM (0.39; 0.16–0.97). **Conclusions:** A total-body CMN count >15, ≥4 iris freckles, and severe sunburn history revealed strong independent associations with increased CM risk. However, associations involving SLC45A2 rs16891982 and IL6 rs1800795 in this Lithuanian case–control study were model-dependent. These findings highlight the potential value of integrating selected genetic markers with phenotypic characteristics and sun-exposure history in the development of personalized CM risk prediction models; however, validation in larger populations is required.

## Simple Summary

Cutaneous melanoma (CM) risk is associated with phenotypic characteristics, sun-exposure history, and genetic markers. Evaluating these factors together may support more accurate identification of individuals at elevated risk. The aim of our case–control study was to assess selected genetic polymorphisms together with skin and iris phenotypic characteristics in relation to CM susceptibility in a Lithuanian population. We observed that the presence of more than 15 common melanocytic nevi, four or more iris freckles, a history of severe sunburn, and the heterozygous SLC45A2 rs16891982 genotype were associated with increased CM odds. Fitzpatrick skin phototypes II and III (compared with phototype IV) and the IL6 rs1800795 Mono G genotype were associated with lower CM odds. These findings highlight the potential value of integrating selected genetic markers with phenotypic characteristics and sun-exposure history in the development of personalized CM risk prediction models; however, validation in larger populations is required.

## 1. Introduction

Cutaneous melanoma (CM) is the most aggressive skin cancer, and its incidence continues to rise worldwide [1]. Registry-based Lithuanian data for 1991–2015 showed rising CM incidence in both sexes, with a higher annual percent change in men (3.9%) than in women (2.3%). Melanoma-related mortality also increased during the same period [2]. More recent epidemiological studies demonstrated the highest increase among individuals older than 60 years, highlighting the growing burden of CM in an aging population [3]. These findings emphasize the need for improved prevention strategies, earlier detection, and more accurate identification of individuals at elevated risk.

Recent case–control studies conducted in Lithuania identified common melanocytic nevi (CMN) [4], Fitzpatrick skin phototypes I and II, light skin and eye color [4,5], and the presence of iris freckles [4] as risk factors for CM. The evidence from our previous study indicates that a blue or grey iris periphery and a blue collarette iris pattern with iris freckles increase the risk of CM more than twofold [4]. Severe sunburns resulting in painful erythema, skin peeling, or blistering were also strongly associated with CM, with each additional episode increasing the risk 2.57-fold [5]. In addition, tanning bed use (≥1 time per year), lack of sunscreen use outdoors on sunny days, and time (≥2 h) spent daily at the beach while vacationing were more often observed in CM cases than in controls [4,5].

Contemporary genomic studies indicate that CM susceptibility is genetically heterogeneous, with genome-wide association studies mapping multiple risk loci across pigmentation, nevogenesis, telomere maintenance, and immune-regulatory pathways [6]. Truderung et al. have described this CM risk as a combination of rare high-penetrance predisposition genes and common low-penetrance variants [7]. Rare predisposing variants have also been identified in melanoma-prone families and high-risk cohorts [8]. Pellegrini et al. have shown that such genetic information becomes most informative when combined with clinical and phenotypic risk factors, since each variant explains only a small part of risk, supporting the evaluation of selected polymorphisms alongside phenotypic traits [9].

Genes involved in melanogenesis and skin pigmentation are among the most consistent genetic determinants of CM risk [6]. Fernandez et al. have identified SLC45A2 (rs26722, rs16891982) as a melanoma-associated melanosomal transporter that supports tyrosinase processing, functioning alongside TYR (rs1126809), the rate-limiting enzyme of melanin synthesis [10]. Variation in these genes influences constitutive pigmentation and the cutaneous response to ultraviolet (UV) exposure, contributing to differences in CM susceptibility [11]. Ibarrola-Villava et al. have demonstrated that the SLC45A2 rs16891982 minor allele is linked to darker pigmentation (Fitzpatrick skin phototypes III–IV; brown, strongly brown, and black eyes; and black and dark brown hair) and to a protective effect against CM [12]. Šepetienė et al. have additionally associated these tyrosinase (TYR) and SLC45A2 variants with skin aging and photoaging-related traits, which motivated their inclusion in the present panel [13].

Inflammatory and immune-regulatory mechanisms also contribute to CM predisposition through cytokine signaling and tumor–host interactions [14]. Ghahartars et al. have shown that interleukin-6 (IL-6) and tumor necrosis factor alpha (TNF-α) enhance the survival and proliferation of melanoma cells [15]. IL-6 further promotes melanoma progression by expanding myeloid-derived suppressor cells through the IL-6/STAT3 axis [16]. Vlaykova et al. have reported that these promoter variants are not themselves predisposing factors for CM, although the IL6 −174G>C genotype and serum IL-6 levels may influence disease progression and survival [17]. TNF-α promoter variants, including rs1800629, have likewise shown inconsistent associations with melanoma susceptibility [18]. Šepetienė et al. previously analyzed these IL6 rs1800795 and TNF-α rs1800629 variants within a skin-aging polymorphism panel, where both contributed to immune-response and skin-protection roles, further supporting their selection for the present study [13].

To our knowledge, this is the first case–control study in Eastern Europe to integrate skin and iris phenotypic characteristics with selected polymorphisms in genes related to melanogenesis and skin pigmentation—TYR rs1126809, SLC45A2 rs26722, and SLC45A2 rs16891982—and inflammatory regulation—IL6 rs1800795 and TNF-α rs1800629—in relation to CM risk. Therefore, this study aimed to evaluate the contribution of these polymorphisms, together with skin and iris phenotypic characteristics, to CM susceptibility and individualized risk assessment.

## 2. Materials and Methods

### 2.1. Study Design

This case–control study analyzed 180 subjects with CM, sourced from the Hospital of the Lithuanian University of Health Sciences Kauno klinikos database. The case group consisted of patients diagnosed with primary CM stages I–IV between 2017 and 2023. The CM stage was classified according to the 8th edition of the American Joint Committee on Cancer (AJCC) staging system [19]. During the study period, 419 CM cases were identified in the hospital database, and 43% of invited patients agreed to participate in the study.

The representative control group consisted of 181 individuals with no personal history of skin cancer or melanoma. These subjects were selected from participants of a Lithuanian population-based study in Kaunas named “*The chronic diseases and their risk factors in the adult population*”. The criteria for inclusion of subjects in the control group of the study were being Caucasian, over 18 years of age, willing to participate in the study, and not diagnosed with CM.

In the control group, women made up 61.8%, and in the case group, 62.8% of the subjects. The higher inclusion of women than men in the study was consistent with the national health statistics data, according to which women made up 63.9% of patients diagnosed with CM [20]. The study was carried out following the Helsinki Declaration principles and local human research guidelines. It was approved by the Kaunas Regional Biomedical Research Ethics Committee (9 June 2021 No. BE-2-66).

### 2.2. Dermatological Examination

All subjects had a comprehensive skin examination. A standardized protocol was used for identifying the total number of CMN [21]. The CMN were described as brown to black pigmented macules or papules that appeared darker than the surrounding skin. Freckles, solar lentigines, or café au lait spots were excluded from the total number of CMN. No distinction between lentigo simplex and junctional melanocytic nevi was sought. Palpable, skin-colored pigmented macules with characteristics of compound or dermal CMN, halo nevi, nevus spilus, congenital nevi, blue nevi, and atypical melanocytic nevi (AMN) were included in the total CMN count. For the statistical analysis of CM risk, the total number of CMN was dichotomized according to the median observed in the combined case and control groups, with 15 nevi used as the cut-off value.

The CMN were counted in 26 anatomical areas, excluding the genital region and buttocks. The size of CMN was measured using a metric mole analyzer with circles ranging from 1 to 12 mm. The CMN were divided by size into three groups: ≤2 mm, 2–5 mm, and ≥5 mm in diameter. As the number of CMN measuring ≤2 mm was high and was not considered relevant for the assessment of CM risk, these lesions were not included in the subsequent analyses presented in this article. For the face, outer surface of the upper arm, and outer surface of the forearm, only the numbers of CMN measuring 2–5 mm in diameter were reported, because CMN measuring ≥5 mm in these anatomical areas were observed only in isolated cases. The AMN were identified using the ABCDE clinical criteria: asymmetry, border irregularity, mixed color, diameter ≥5 mm, and erythema at the margins or elevation of the freckles. The diagnosis of AMN was established if at least three of the five clinical criteria were fulfilled, and these lesions were included in the total number of CMN. Dermatoscopy was used to distinguish AMN from other pigmented freckles.

To ensure assessment reliability, the skin examination was performed by three medical doctors trained by an experienced dermatologist (S.V.). The accuracy of CMN and/or AMN assessment was evaluated in a pilot substudy by one of the investigators (S.V.). The level of agreement in counting the number of CMN on the total body between the trained medical doctors and the experienced dermatologist differed by less than 5%.

### 2.3. Ophthalmological Examination

Iris freckling was evaluated using a standardized imaging protocol [22] and is described in detail in our previous study [4], in which high-resolution images of the anterior segment of each participant’s eyes were obtained for analysis. High-resolution photographs were obtained using a BQ 900 slit lamp equipped with an IL 900 photographic module (Haag-Streit, Bern, Switzerland). Images were captured at 16× magnification with the slit beam intensity set to level 4 and the background illumination set to level 3. A diffuser was used and the light source was angled at 45° laterally to maximize illumination of the iris while minimizing glare. Special attention was given to positioning the reflection of the light source within the pupil area or at a location that minimally obscured iris pigmented freckles. To enhance photo quality and reduce pupillary reaction, several iris photographs were taken during twilight, prior to pharmacologic mydriasis. Blurry or poorly illuminated images were excluded.

Iris freckles were assessed by two experienced ophthalmologists. Each image was reviewed separately by both investigators, who were blinded to participant group allocation. All photographs were anonymized and coded prior to evaluation. After the assessment, the codes were decrypted and the iris freckle counts were assigned to the corresponding participants.

The number of freckles present on each iris was recorded, and a mean value for both eyes was calculated for each subject [4]. Based on the median value of the iris freckle sum across the study population, a binary categorical variable was created: participants with ≤3 freckles and those with ≥4. Other ocular features—such as pigmented or nonpigmented iris nevi, Lisch nodules, partial heterochromia, and iris melanoma—were not included in the number of freckles.

### 2.4. Questionnaire About Habits of Sun Exposure and Skin Phototype

All participants completed a questionnaire regarding their habits related to sun exposure. The survey included questions about the frequency and duration of time spent in the sun, the use of protective measures such as sunscreen and sunglasses, and the extent to which they sought shade or wore protective clothing [5]. Additionally, the subjects were asked whether they had experienced severe sunburns, defined as painful erythema, skin peeling, or blistering after UV exposure. Participants in the CM group were specifically instructed to report their sun-exposure habits during the period preceding the diagnosis of CM, thereby excluding any behavioral changes that may have occurred after diagnosis. For individuals in the CM group, sun-exposure behavior was assessed with a particular focus on the year preceding the confirmed diagnosis of CM, whereas for the control group, it was assessed over a comparable recent period. The subjects were asked about their skin sensitivity to the sun. Based on the answers, their skin phototype was classified according to Fitzpatrick: skin phototype I (pale white skin, always burns and never tans); phototype II (light skin, usually burns, tans minimally); phototype III (medium-light skin, mild burns, tans moderately); and phototype IV (light brown skin, rarely burns, tans easily), respectively [23].

### 2.5. Determination of Genotypes

This part of the study was performed in the Laboratory of Molecular Cardiology at the Lithuanian University of Health Sciences. Genomic DNA was extracted from whole blood samples using the salting-out genomic DNA extraction method. DNA quality and concentration were assessed using a Nanodrop 1000 spectrophotometer (Thermo Fisher Scientific, Waltham, MA, USA).

The genotyping of TYR (rs1126809, assay ID: C___175675702_10), SLC45A2 (rs26722, assay ID: C___2390575_1_; rs16891982, assay ID: C___2842665_10), IL6 (rs1800795, assay ID: C___1839697_20), and TNF-α (rs1800629, assay ID: C___7514879_10) was performed according to the manufacturer’s instructions using TaqMan chemistry on a QuantStudio 5 real-time PCR thermocycler (Applied Biosystems, Waltham, MA, USA).

The selection of single nucleotide polymorphisms (SNPs) analyzed in this study was based on a previously published panel of 15 skin-related genetic variants [13]. SNP selection was based on their associations with skin resistance to UV-induced damage (rs1126809, rs26722, rs16891982) and immune response modulation (rs1800795, rs1800629), both of which are important for CM development [12,24,25,26,27,28]. To specify SNPs potentially associated with CM, a literature review was conducted using the PubMed NCBI database. Keywords “*melanoma, melanocyte, skin aging*” and individual SNP ID numbers from the previous study were used. Filters including open-access and full-text articles published within the last five years were applied. A total of 42 articles were found. Based on the frequency of reported associations with CM or skin pigmentation and relevance to European populations, five SNPs were selected for final analysis. Results are described based on SNP allele sequences and represented as wild-type, heterozygous, and minor genotypes. Since no minor homozygous genotype of rs1800795 was observed in the study population, genotypes were classified as Mono G or Mono C.

### 2.6. Data Analysis

Data entry was carried out using Microsoft Office Excel 2021. Statistical analysis was performed using IBM SPSS Statistics, version 30.0.

The Kolmogorov–Smirnov test was applied to assess the normality of quantitative data, and Levene’s test was used to assess the homogeneity of variance. Quantitative variables were characterized by the mean and standard deviation (SD) or the median including the minimum and maximum values (range) and were compared between two groups using Student’s t/Mann–Whitney tests depending on the distribution of the data. Qualitative variables were described by frequency (*n*) and the relative frequency (%) and were compared using Chi-square (X^2^)/Fisher’s exact tests in the comparative groups.

Multivariable binary logistic regression analysis was used to estimate age- and sex-adjusted odds ratios (OR) with 95% confidence intervals (CI) for each variable separately in relation to CM. Subsequently, a multivariable logistic regression model was constructed including variables with statistically significant odds ratios (OR) for CM in the individual analyses. Statistical significance was defined as *p* < 0.05.

## 3. Results

Participants in the case group had a mean age of 59.56 (SD = 12.51) years, compared with 57.61 (SD = 7.97) years in the control group. No statistically significant differences in age or sex distribution were observed between the groups (Table 1). Several phenotypic characteristics varied notably between the groups. Skin color distribution differed significantly, with light skin more frequently reported in cases (48.6%) than in controls (29.7%). Iris freckles were present in 94.8% of individuals in the case group and in 48.6% of those in the control group. The median number of CMN measuring >2 mm in diameter was 24 and 5 in the case and control groups, respectively (*p* < 0.001). The total count of CMN >2 mm was greater in the case group, with 66.9% of individuals having more than 15 nevi, compared to 28.2% in the control group. Higher numbers of CMN on the face and outer surface of the upper arm were observed in cases than in controls. On the outer surface of the lower arm, only the number of CMN measuring 2–5 mm was significantly higher in cases, whereas the total CMN count did not differ significantly between the groups. History of severe sunburns was more common in the case group than in controls. Use of sunscreen at the beach also differed significantly, with regular use reported more frequently by controls than by cases (Table 1). The distribution of the SLC45A2 rs26722 polymorphism showed a significant difference, with the wild-type genotype more commonly observed in cases (99.4%) than in controls (96.1%) (*p* < 0.05). In contrast, no statistically significant between-group differences in genotype distributions were found for the other analyzed polymorphisms, including TYR (rs1126809), SLC45A2 (rs16891982), IL6 (rs1800795), and TNF-α (rs1800629). Characteristics of the evaluated CM risk factors are presented in Table 1.

Binary logistic regression analysis was used to estimate age- and sex-adjusted odds ratios for individual variables, identifying several factors associated with increased odds of CM (Table 2). Neither sex (OR = 0.96, 95% CI: 0.63–1.47) nor age (OR = 1.02, 95% CI: 1.00–1.04) was statistically significant predictors of increased CM risk. Iris freckles were most strongly linked to CM. The presence of more than 15 CMN >2 mm in diameter on the body was associated with increased odds of CM. Additionally, each additional CMN >2 mm in diameter significantly increased odds of CM. Compared to individuals with olive skin, those with medium skin had significantly lower odds of CM. Similarly, Fitzpatrick phototype II was associated with lower CM odds compared with phototype IV. A history of severe sunburn was associated with increased odds of CM. Not using sunscreen or using it only occasionally was associated with higher odds at the beach and showed a tendency toward higher odds on sunny summer days. No significant associations were observed for the examined genetic variants in TYR, SLC45A2, IL6, or TNF-α after adjustment for age and sex (Table 2).

A multivariable logistic regression model with backward elimination including selected phenotypic and genetic variables identified several independent predictors of CM (Table 3). Participants with more than 15 CMN had markedly increased CM odds (OR = 7.89; 95% CI: 4.29–14.53), as did those with four or more iris freckles (OR = 3.70; 95% CI: 2.04–6.74). A history of severe sunburn involving peeling or blistering was also associated with elevated risk (OR = 2.76; 95% CI: 1.50–5.07). Compared with Fitzpatrick skin phototype IV, phototypes III (OR = 0.28; 95% CI: 0.11–0.70) and II (OR = 0.35; 95% CI: 0.15–0.82) were associated with significantly lower odds, while no association was observed for phototype I. Hair color was not significantly related to CM risk. The heterozygous SLC45A2 rs16891982 genotype was linked to increased odds (OR = 6.64; 95% CI: 1.48–29.83), whereas the IL6 rs1800795 Mono G variant was associated with decreased risk (OR = 0.39; 95% CI: 0.16–0.97). Full regression results are provided in Table 3.

## 4. Discussion

In the present study, we investigated selected genetic polymorphisms related to pigmentation and inflammatory signaling, including SLC45A2 rs26722, SLC45A2 rs16891982, TYR rs1126809, IL6 rs1800795, and TNF-α rs1800629, together with phenotypic characteristics, including skin color, Fitzpatrick skin phototype, hair color, CMN count, iris freckles, severe sunburn history, and sunscreen use. Overall, a CMN count > 15, a sum of iris freckles ≥ 4, and a history of severe sunburns showed higher odds ratios in cases compared to controls. Of the selected genetic polymorphisms, only IL6 (rs1800795) Mono G and SLC45A2 (rs16891982) heterozygous genotypes were associated with CM risk in the multivariable binary logistic regression model. Previous Lithuanian case–control studies similarly identified iris freckles and severe sunburns as factors associated with CM risk [4,5]. In contrast, the findings for the selected polymorphisms were less consistent and should be considered model-dependent additional findings rather than stable independent markers of CM risk. The distribution of the SLC45A2 rs26722 polymorphism showed a significant difference, with the wild-type genotype more commonly observed in cases than in controls. In contrast, no statistically significant associations were found for the other analyzed polymorphisms, including TYR (rs1126809), SLC45A2 (rs16891982), IL6 (rs1800795), and TNF-α (rs1800629). Binary logistic regression analysis showed that no significant associations were observed for the examined genetic variants in TYR, SLC45A2, IL6, or TNF-α after adjustment for age and sex. A multivariable logistic regression model with backward elimination including selected phenotypic and genetic variables identified several independent predictors of CM. The heterozygous SLC45A2 rs16891982 genotype was linked to increased odds, whereas the IL6 rs1800795 Mono G variant was associated with decreased odds.

The five SNPs investigated in this study represent a limited candidate-gene panel and do not capture the broader genetic architecture of CM susceptibility. Their relevance to CM also differs, as SLC45A2 and TYR variants are primarily linked to pigmentation-related pathways, whereas IL6 and TNF-α variants are involved in inflammatory regulation, with less consistent evidence for direct associations with primary CM susceptibility.

Large GWAS-based studies have shown that the inherited component of CM risk is genetically heterogeneous and involves multiple risk loci, supporting the view that individual candidate polymorphisms usually explain only a limited part of CM risk [6]. In the present Lithuanian study, the distribution of SLC45A2 rs26722 differed between the case and control groups, with the wild-type genotype being more frequent in the CM group. However, this difference did not translate into a statistically significant odds ratio in the age- and sex-adjusted model. In contrast, SLC45A2 rs16891982 showed no significant between-group difference in the descriptive comparison, but its heterozygous genotype was associated with increased odds of CM in the multivariable model. These findings suggest that the contribution of SLC45A2 variants may depend on the statistical model and should be interpreted cautiously, particularly given the low frequency of some genotype categories.

Among the studied pigmentation-related polymorphisms, SLC45A2 rs26722, SLC45A2 rs16891982, and TYR rs1126809 were biologically relevant candidates because SLC45A2 and TYR participate in melanogenesis and pigmentation-related pathways. Previous studies have linked selected variants in these genes to CM susceptibility, supporting their relevance to CM risk [10,12,29]. More recently, Balogh et al. specifically discussed TYR rs1126809 and suggested that variation around this locus may show population- and haplotype-dependent patterns relevant to CM risk [30]. In the present study, TYR rs1126809 was not significantly associated with CM risk.

IL6 rs1800795 and TNF-α rs1800629 are involved in inflammatory signaling and tumor–host interactions, but available evidence does not consistently support inflammatory promoter variants as stable markers of primary CM susceptibility. Vlaykova et al. evaluated IL6 −174G > C and TNF-α −308G > A promoter polymorphisms in patients with CM and reported that these variants were not associated with increased susceptibility to CM, although IL6-related variation and serum IL-6 levels may be more relevant to disease progression than to primary susceptibility [17]. In a meta-analysis focused on IL6 polymorphisms and skin cancer susceptibility, Guo et al. found no clear overall association between IL6 variants, including rs1800795, and skin cancer risk [31]. Similarly, Liu et al. reported that TNF-α promoter polymorphisms, including rs1800629, were not consistently associated with susceptibility to CM and other skin cancers in pooled studies [18]. In the present study, TNF-α rs1800629 was not significantly associated with CM risk, whereas IL6 rs1800795 Mono G was associated with decreased odds of CM in the multivariable model. This finding may indicate a model-dependent association, but it should be interpreted with caution and confirmed in larger CM-specific studies.

In our study, Fitzpatrick skin phototypes II and III were associated with lower odds of CM compared with phototype IV, whereas phototype I showed no significant association with CM risk. This unexpected finding may be related to the relatively small number of participants with phototype IV, which was used as the reference group, as well as to the phenotypic homogeneity of the Lithuanian study population. This differs from the findings of Kulichová et al., who reported a higher likelihood of CM among individuals with phototypes I and II than among those with phototype III in a case–control study conducted in the Czech Republic and Germany [32].

Medium skin color was associated with lower odds of CM than olive skin color. This finding contrasts with that of Landi et al., who reported higher odds of CM among individuals with medium and light skin color than among those with dark/olive skin in an Italian case–control study [33]. Similarly, Ghiasvand et al. found that medium and light skin color were associated with a higher CM risk than dark skin color in a population-based cohort study from Norway [34]. This difference in the present study may be related to the small number of participants with olive skin color, as olive skin color was used as the reference group.

Regarding hair color, in contrast to our findings, Lewandowska et al. reported that blond and red hair were associated with a higher CM risk than dark brown or black hair in a Polish case–control study [35].

A strong association between iris freckles and CM risk was observed in our study. Individuals with iris freckles had a higher (nearly 19-fold higher odds) likelihood of CM. This finding is consistent with a regional study from South-Eastern Europe, where Grigore et al. identified iris pigmentation patterns, including freckles, as phenotypic markers of skin cancer risk, although their study was not limited to CM [36]. Our previous study by Šemeklis et al. [4] also showed a higher prevalence of iris freckles among CM patients [4]. Importantly, this study further suggests that not only the presence but also a higher number of iris freckles, particularly four or more, is associated with increased CM risk. Similar findings have been reported in an Australian case–control study by Laino et al., in which the presence of three or more iris pigmented lesions was associated with increased odds of CM [37].

In a multicenter European case–control study (including regionally closer participants from Finland and Poland), de Vries et al. confirmed the presence of CMN and AMN as risk factors for CM [38]. Similarly, in a Swedish case–control study, Augustsson et al. observed that the CMN count in patients with CM was nearly twice as high as that observed in controls [39], similar to our study results. In line with our previous studies including our earlier work [4,5,40], we observed that a higher number of CMN, particularly those ≥ 2 mm, was significantly associated with increased CM risk.

Our study identified a high melanocytic nevus count and iris freckles as independent CM risk factors. In particular, a high CMN burden may also reflect an underlying hereditary predisposition, such as Familial Atypical Multiple Mole Melanoma (FAMMM) syndrome, which is strongly associated with germline CDKN2A mutations. The Northern European p16-Leiden CDKN2A variant is a well-recognized example linking CM susceptibility with an increased risk of pancreatic carcinoma [41]. Therefore, future studies and clinical practice settings should pay particular attention to patients with multiple and/or dysplastic melanocytic nevi, as they may benefit from genetic counseling, CDKN2A testing, and surveillance for pancreatic and other internal malignancies.

In our study, a history of severe sunburn was significantly associated with increased CM risk. This finding is consistent with previous case–control studies in the Eastern European region [35,42]. Similarly, Lewandowska et al. observed a significant association between sunburn episodes before the age of 30 and CM risk, supporting the role of early-life intermittent intense UV exposure in CM development [35].

However, our findings regarding sunscreen use differed depending on the context of use. Not using sunscreen or using it occasionally on the beach was linked to a higher CM risk, while not using sunscreen on sunny summer days showed no clear association with CM. Context-dependent results were also reported in Czech case–control studies. Rajská et al. found that occasional sunscreen use was associated with lower CM risk, while regular use every time showed no association with the risk of CM [43]. Vranova et al. identified inadequate sunscreen use in adulthood and all-day sun exposure, including peak UV hours from 11 AM to 3 PM, as relevant CM risk factors [42]. However, recent case–control studies from Eastern European populations that directly distinguish sunscreen use on the beach from sunscreen use during routine sunny-day exposure remain limited. Therefore, direct comparison with previous regional studies is restricted by differences in exposure definitions and questionnaire design.

Several methodological limitations should be acknowledged when interpreting the results of this study. The relatively small sample size may have reduced the statistical power to detect modest genotype-related effects. The genetic analysis was limited to a predefined panel of five candidate SNPs and therefore does not represent the full spectrum of genetic susceptibility to CM. Furthermore, our study involved a relatively homogeneous Lithuanian population. Future multicenter studies are needed to assess the associations of phenotypic characteristics, particularly iris freckles, and a broader range of genetic variants with CM risk in more diverse populations, including individuals with Fitzpatrick skin phototypes IV–VI. Assessing body fat percentage alongside BMI could be a valuable addition to future studies, allowing a more detailed characterization of participants’ body composition.

Taken together, our findings indicate that selected genetic polymorphisms, SLC45A2 rs26722, SLC45A2 rs16891982, TYR rs1126809, IL6 rs1800795, and TNF-α rs1800629, may contribute to CM risk stratification, but their associations with CM risk were less consistent than light skin color, presence of iris freckles, count of CMN, history of severe sunburn, and use of sunscreen. Rather, the results suggest that isolated candidate polymorphisms may provide limited standalone predictive value and may be more informative when considered together with phenotypic and UV-related risk factors.

From a clinical perspective, our findings suggest that a simple assessment of phenotypic characteristics, particularly iris freckles and total melanocytic nevus count, during routine dermatological examinations may help identify individuals at elevated risk of CM. Given that these readily accessible clinical markers can be evaluated during routine patient visits without additional costs or equipment, they may serve as practical tools for risk stratification, improving personalized screening strategies and supporting earlier detection of CM.

Future studies involving larger, genetically diverse integrated models are needed to clarify the contribution of these polymorphisms to CM risk.

## 5. Conclusions

In conclusion, this case–control study showed that CM susceptibility within the Lithuanian population was more consistently associated with observable phenotypic traits and sun-exposure history than with selected candidate-gene polymorphisms. Specifically, a total-body melanocytic nevus count >15, the presence of ≥4 iris freckles, and a history of severe sunburn emerged as robust, independent predictors of increased CM risk. While the evaluated single nucleotide polymorphisms (TYR rs1126809, SLC45A2 rs26722, SLC45A2 rs16891982, IL6 rs1800795, and TNF-α rs1800629) are biologically relevant to pigmentation and inflammatory pathways, the associations observed for SLC45A2 rs16891982 and IL6 rs1800795 were model-dependent and require validation in larger populations. These findings may inform the future development of personalized CM risk prediction models integrating selected genetic markers with phenotypic characteristics and sun-exposure history, while underscoring the importance of severe sunburn prevention.

## Figures and Tables

**Table 1:** Characteristics of study participants in the cutaneous melanoma case and control groups.

Characteristics	Control Group *N* = 181	Case Group *N* = 180	*p*-Value
Sex, % (*n*)			0.914
Male	38.1 (69)	37.2 (67)	
Female	61.8 (112)	62.8 (113)	
Total	100.0 (181)	100.0 (180)	
Age (years), mean (SD)			
Male	57.97 (8.2)	60.70 (13.5)	0.158
Female	57.38 (7.85)	58.88 (11.89)	0.265
Total	57.61 (7.97)	59.56 (12.51)	0.078
Height (cm), mean (SD)	169.35 (9.33)	169.44 (9.81)	0.930
Weight (kg), mean (SD)	82.2 (17.5)	85.4 (18.54)	0.094
BMI (kg/m^2^), mean (SD)	28.64 (5.64)	29.79 (6.42)	0.073
**Skin color, % (*n*)**			**<0.001**
**Light**	**29.7 (52)**	**48.6 (87)**	
**Medium**	**65.7 (115)**	**40.8 (73)**	
**Olive**	**4.5 (8)**	**10.6 (19)**	
Skin phototype (Fitzpatrick scale), % (*n*)			0.051
Phototype I	15.3 (27)	21.7 (39)	
Phototype II	39.8 (70)	36.1 (65)	
Phototype III	30.7 (54)	21.1 (38)	
Phototype IV	14.2 (25)	21.1 (38)	
Hair color, % (*n*)			0.153
Light brown	52.0 (91)	62.0 (111)	
Dark brown	33.1 (58)	25.1 (45)	
Black	14.9 (26)	12.8 (23)	
**Presence of iris freckles, % (*n*)**			**<0.001**
**Yes**	**48.6 (85)**	**94.8 (165)**	
**No**	**51.4 (90)**	**5.2 (9)**	
**Average number of iris freckles, median (range)**	**0.5 (0–19.5)**	**2.5 (0–30)**	**<0.001**
**Number of total-body CMN, median (range)**			
**>2 mm in diameter**	**5.0 (0–149)**	**24.0 (0–216)**	**<0.001**
**Number of CMN >2 mm in diameter, % (*n*)**			
**15 or less**	**71.8 (130)**	**33.1 (59)**	**<0.001**
**More than 15**	**28.2 (51)**	**66.9 (119)**	
**Number of CMN on face, median (range)**			
**Diameter 2–5 mm**	**0 (0–4)**	**0 (0–10)**	**<0.001**
**Total**	**0 (0–14)**	**1.0 (0–24)**	**<0.001**
**Number of CMN on outer surface of upper arm, mean (SD)**			
**Diameter 2–5 mm**	**1.40 (3.667)**	**3.52 (4.718)**	**<0.001**
**Total**	**6.62 (8.561)**	**10.19 (11.467)**	**<0.001**
**Number of CMN on outer surface of lower arm, mean (SD)**			
**Diameter 2–5 mm**	**0.34 (1.047)**	**1.57 (2.701)**	**<0.001**
Total	3.53 (5.341)	5.65 (17.725)	0.126
**Had severe sunburn during the last year, % (*n*)**			**<0.001**
**Yes**	**69.3 (122)**	**90.4 (161)**	
**No**	**30.7 (54)**	**9.6 (17)**	
Use of sunscreen outside on sunny days, % (*n*)			0.066
Do not use sunscreen or use occasionally	67.6 (119)	76.4 (133)	
Uses every time	32.4 (57)	23.6 (41)	
**Use of sunscreen at the beach, % (*n*)**			**0.013**
**Do not use sunscreen or use occasionally**	**40.7 (68)**	**54.5 (84)**	
**Uses every time**	**59.3 (99)**	**45.5 (70)**	
Corresponding gene with investigated polymorphism			0.348
TYR (rs1126809), % (*n*)			
Wild-type genotype	59.1 (107)	53.1 (95)	
Heterozygous genotype	34.8 (63)	37.4 (67)	
Minor genotype	6.1 (11)	9.5 (17)	
**Corresponding gene with investigated polymorphism**			**0.033**
**SLC45A2 (rs26722), % (*n*)**			
**Wild-type genotype**	**96.1 (174)**	**99.4 (179)**	
**Heterozygous genotype**	**3.9 (7)**	**0.6 (1)**	
Corresponding gene with investigated polymorphism			0.086
SLC45A2 (rs16891982), % (*n*)			
Wild-type genotype	94.5 (171)	98.3 (177)	
Heterozygous genotype	5.5 (10)	1.7 (3)	
Corresponding gene with investigated polymorphism			0.298
IL6 (rs1800795), % (*n*)			
Heterozygous genotype	51.9 (94)	55.6 (100)	
Mono C	30.9 (56)	23.9 (43)	
Mono G	17.1 (31)	20.6 (37)	
Corresponding gene with investigated polymorphism			0.099
TNF-α (rs1800629), % (*n*)			
Wild-type genotype	75.1 (136)	77.2 (139)	
Heterozygous genotype	24.3 (44)	19.4 (35)	
Minor genotype	0.6 (1)	3.3 (6)	

Bold formatting indicates statistically significant results (*p* < 0.05); *n*—the number of individuals or observations in the group; SD—standard deviation; mm—millimeters in diameter; *p*-Value < 0.05; BMI—body mass index; %—percentage; cm—centimeters; kg—kilograms; kg/m^2^—kilograms per square meter.

**Table 2:** Logistic regression models of individual variables associated with cutaneous melanoma.

Variable	Odds Ratio *	95% Confidence Interval		*p*-Value
		Lower Bound	Upper Bound	
Body mass index (kg/m^2^)	1.03	0.99	1.06	0.183
**Skin color, olive (reference)**				
Light	0.66	0.27	1.64	0.370
**Medium**	**0.26**	**0.11**	**0.63**	**0.003**
**Skin phototype (Fitzpatrick scale), phototype IV (reference)**				
Phototype I	1.16	0.56	2.43	0.689
**Phototype II**	**0.50**	**0.26**	**0.96**	**0.037**
Phototype III	0.67	0.37	1.23	0.198
Hair color, black (reference)				
Light brown	1.41	0.75	2.67	0.290
Dark brown	0.92	0.46	1.83	0.804
**Presence of pigmented iris freckles**	**18.95**	**9.08**	**39.55**	**<0.001**
**Number of CMN**				
**>2 mm in diameter**	**1.04**	**1.02**	**1.05**	**<0.001**
**Number of CMN >2 mm in diameter, 15 or less (reference)**				
**More than 15**	**5.06**	**3.22**	**7.95**	**<0.001**
**Had severe sunburn during the last year**	**2.65**	**1.70**	**4.15**	**<0.001**
Use of sunscreen on **sunny summer days,** uses every time in the sun (reference)				
Do not use sunscreen or use occasionally	1.54	0.94	2.53	0.088
**Use of sunscreen at the beach, uses every time (reference)**				
**Do not use sunscreen or use occasionally**	**1.76**	**1.09**	**2.85**	**0.020**
Corresponding gene with investigated polymorphism TYR (rs1126809), wild-type genotype (reference)				
Heterozygous genotype	1.22	0.78	1.90	0.381
Minor genotype	1.62	0.72	3.67	0.244
Corresponding gene with investigated polymorphism SLC45A2 (rs26722), wild-type genotype (reference)				
Heterozygous genotype	0.13	0.02	1.08	0.059
Corresponding gene with investigated polymorphism SLC45A2 (rs16891982), wild-type genotype (reference)				
Heterozygous genotype	0.29	0.08	1.08	0.065
Corresponding gene with investigated polymorphism IL6 (rs1800795), heterozygous genotype (reference)				
Mono C	0.68	0.42	1.12	0.130
Mono G	1.04	0.59	1.82	0.903
Corresponding gene with investigated polymorphism TNF-α (rs1800629), wild-type genotype (reference)				
Heterozygous genotype	0.81	0.49	1.34	0.412
Minor genotype	5.47	0.64	46.58	0.120

* Odds ratio adjusted by age and sex; bold formatting indicates statistically significant results (*p* < 0.05); mm—millimeters in diameter; kg/m^2^—kilograms per square meter.

**Table 3:** Multivariable binary logistic regression model identifying phenotypic and genetic predictors of cutaneous melanoma.

Variable	Odds Ratio *	95% Confidence Interval		*p*-Value
		Lower Bound	Upper Bound	
**Skin phototype (Fitzpatrick scale), phototype IV (reference)**				
**III skin phototype**	**0.28**	**0.11**	**0.70**	**0.007**
**II skin phototype**	**0.35**	**0.15**	**0.82**	**0.016**
I skin phototype	0.81	0.29	2.26	0.692
Hair color, black (reference)				
Light brown hair	1.22	0.50	2.98	0.656
Dark brown hair	0.58	0.22	1.55	0.279
**Sum of iris freckles, ≤3 (reference)**				
**Sum of iris freckles ≥4**	**3.70**	**2.04**	**6.74**	**<0.001**
**Number of CMN >2 mm in diameter, 15 or less (reference)**				
**More than 15**	**7.89**	**4.29**	**14.53**	**<0.001**
**History of sunburns, did not have severe sunburn (reference)**				
**Had one or more severe sunburns**	**2.76**	**1.50**	**5.07**	**<0.001**
**SLC45A2 (rs16891982), wild-type genotype (reference)**				
**SLC45A2 (rs16891982) heterozygous genotype**	**6.64**	**1.48**	**29.83**	**0.014**
**IL6 (rs1800795), heterozygous genotype (reference)**				
IL6 (rs1800795) Mono C	0.96	0.43	2.12	0.916
**IL6 (rs1800795) Mono G**	**0.39**	**0.16**	**0.97**	**0.042**

* Odds ratio adjusted by age and sex; bold formatting indicates statistically significant results (*p* < 0.05).

## Data Availability

The data presented in this study are available on request from the corresponding author.

## Abbreviations

The following abbreviations are used in this manuscript:

LSMU	Lithuanian University of Health Sciences
CM	Cutaneous Melanoma
CMN	Common Melanocytic Nevi
UV	Ultraviolet
TYR	Tyrosinase
IL-6	Interleukin-6
TNF-α	Tumor Necrosis Factor Alpha
AJCC	American Joint Committee on Cancer
AMN	Atypical Melanocytic Nevi
SNPs	Single Nucleotide Polymorphisms
IBM SPSS	International Business Machines Statistical Package for the Social Sciences
SD	Standard Deviation
OR	Odds Ratios
CI	Confidence Intervals
X^2^	Chi-Square
BMI	Body Mass Index
